# 2-(4-Dimethyl­amino-2-hydroxy­benzoyl)benzoic acid methanol solvate

**DOI:** 10.1107/S1600536808038403

**Published:** 2008-11-22

**Authors:** Jin-Chun Guo, Qing-Hao Liu, Jin-Ying Guo, Mei-Zhu Chen, Rui-Wang Zhang

**Affiliations:** a Qinghai Institute of Salt Lakes, Chinese Academy of Sciences, Xining 810008, People’s Republic of China; b School of Chemical Engineering and Technology, Tianjin University, Tianjin 300072, People’s Republic of China; c College of Food and Bioengineering, He’nan University of Science and Technology, Luoyang 471003, People’s Republic of China

## Abstract

In the title compound, C_16_H_15_NO_4_·CH_4_O, the dihedral angle between the benzene rings is 75.21 (5)°. The structure is stabilized by an intra­molecular O—H⋯O inter­action [O⋯O = 2.589 (2) Å]. The solvent mol­ecule links symmetry-related mol­ecules of the complex *via* hydrogen bonds with O⋯O separations of 2.631 (2) and 2.815 (2) Å. C—H⋯O hydrogen bonds are also present.

## Related literature

For a related structure, see: Yan *et al.* (2006 ▶). For synthetic applications, see: Hellmut & Lamm (1977 ▶); Minru *et al.* (1977 ▶); Yojiro *et al.* (1992 ▶); Lee *et al.* (1998 ▶); Luo *et al.* (1994 ▶).
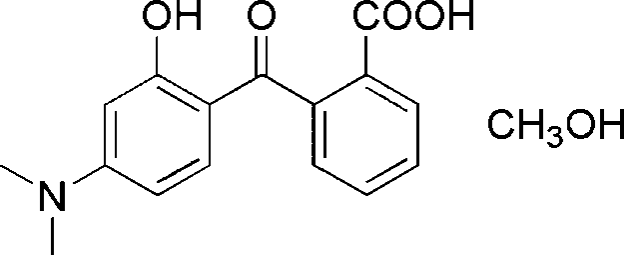


## Experimental

### 

#### Crystal data

C_16_H_15_NO_4_·CH_4_O
*M* *_r_* = 317.33
Triclinic, 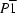

*a* = 7.1438 (14) Å
*b* = 7.3021 (15) Å
*c* = 16.613 (3) Å
α = 83.92 (3)°
β = 80.21 (3)°
γ = 64.94 (3)°
*V* = 773.0 (3) Å^3^
*Z* = 2
Mo *K*α radiation
μ = 0.10 mm^−1^
*T* = 113 (2) K
0.20 × 0.18 × 0.12 mm

#### Data collection

Rigaku Saturn CCD area-detector diffractometer
Absorption correction: multi-scan (*SADABS*; Sheldrick, 1996 ▶) *T*_min_ = 0.980, *T*_max_ = 0.988
12548 measured reflections
3528 independent reflections
2679 reflections with *I* > 2σ(*I*)
*R*_int_ = 0.034

#### Refinement

*R*[*F* ^2^ > 2σ(*F* ^2^)] = 0.036
*wR*(*F* ^2^) = 0.109
*S* = 1.06
3528 reflections
220 parameters
H atoms treated by a mixture of independent and constrained refinement
Δρ_max_ = 0.31 e Å^−3^
Δρ_min_ = −0.25 e Å^−3^

### 

Data collection: *CrystalClear* (Rigaku/MSC, 2005 ▶); cell refinement: *CrystalClear*; data reduction: *CrystalStructure* (Rigaku/MSC, 2005 ▶); program(s) used to solve structure: *SHELXS97* (Sheldrick, 2008 ▶); program(s) used to refine structure: *SHELXL97* (Sheldrick, 2008 ▶); molecular graphics: *SHELXTL* (Sheldrick, 2008 ▶); software used to prepare material for publication: *SHELXTL*.

## Supplementary Material

Crystal structure: contains datablocks global, I. DOI: 10.1107/S1600536808038403/pv2121sup1.cif

Structure factors: contains datablocks I. DOI: 10.1107/S1600536808038403/pv2121Isup2.hkl

Additional supplementary materials:  crystallographic information; 3D view; checkCIF report

## Figures and Tables

**Table 1:** Hydrogen-bond geometry (Å, °)

*D*—H⋯*A*	*D*—H	H⋯*A*	*D*⋯*A*	*D*—H⋯*A*
O1—H1⋯O2	0.92 (2)	1.75 (2)	2.589 (2)	151 (1)
O4—H4⋯O5^i^	0.94 (2)	1.70 (2)	2.631 (2)	168 (2)
O5—H5⋯O2	0.87 (2)	1.95 (2)	2.815 (2)	178 (2)
C7—H7*B*⋯O3^ii^	0.98	2.54	3.463 (2)	156
C13—H13⋯O5^iii^	0.95	2.53	3.331 (2)	142

Symmetry codes: (i) 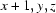; (ii) 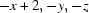; (iii) 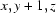.

**Fig. 1.**
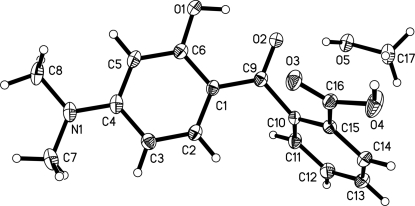
A view of the title compound showing the atom-labelling scheme. Displacement ellipsoids are drawn at the 50% probability level (arbitrary spheres for the H atoms).

**Fig. 2.**
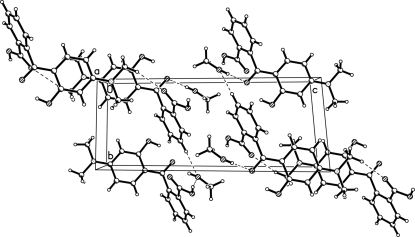
The unit cell packing of (I); dashed lines indicate hydrogen-bond interactions.

## supplementary crystallographic information

### Comment

The title compound, (I), is an intermediate in the synthesis of rhodamine derivatives (Hellmut & Lamm, 1977; Minru *et al.*, 1977; Yojiro *et al.*, 1992; Lee *et al.*, 1998). 2-Carboxyl-4'-diethylamino-2'-hydroxybenzophenone was synthesized (Luo *et al.*, 1994) from 3-diethylaminophenol and phthalic anhydride in toluene with the same reaction mechanism as the title compound. In the present paper, the title compound has been synthesized and the crystal structure of (I) is reported.

The bond distances and bond angles in (I) (Fig. 1) are similar to the corresponding dimensions reported for a closely related structure, 2-[4-(diethylamino)-2-hydroxybenzoyl]-3,4,5,6-tetrafluorobenzoic acid (Yan *et al.*, 2006). The 2-hydroxy-4-dimethylaminobenzoyl and *o*-benzoic acid moieties in (I) are each essentially planar and the angle between two planes is 75.21 (5)°. There are intermolecular O—H···O hydrogen bonds involving the solvent and the complex molecules stabilizing the structure (Table 1 and Fig. 2). The structure also contains an intramolecular interaction of the type O—H···O (O···O = 2.589 (2) Å) and non-classical hydrogen bonds of the type C—H···O.

### Experimental

A solution of 3-dimethylamino phenol (4.11 g, 30.0 mmol) and phthalic anhydride (4.66 g, 31.5 mmol) in toluene (30 ml) was refluxed for 3 h. The solution was cooled to room temperature and the precipitate was collected to afford the title compound (yield = 75.6%). The crude product was purified by silica-gel chromatography (methanol–dichloromethane, 1:50). Crystals suitable for X-ray diffraction were obtained by slow evaporation of a solution in methanol and dichloromethane (5:1).

### Refinement

The O-bound H atoms were located in a difference map and their coordinates were refined with *U*_iso_(H) = 1.5*U*_eq_(O). The methyl and aryl H atoms were constrained to ideal geometry with C—H distances of 0.95 and 0.98 Å, and *U*_iso_(H) = 1.5 and 1.2*U*_eq_(C), respectively, and each methyl group was allowed to rotate freely about its C—C bond.

### Crystal data

**Table:**

C_16_H_14_NO_4_·C_1_H_4_O_1_	*Z* = 2
*M**_r_* = 317.33	*F*_000_ = 336
Triclinic, *P*1	*D*_x_ = 1.363 Mg m^−^^3^
Hall symbol: -P 1	Mo *K*α radiation λ = 0.71073 Å
*a* = 7.1438 (14) Å	Cell parameters from 2451 reflections
*b* = 7.3021 (15) Å	θ = 2.5–27.5º
*c* = 16.613 (3) Å	µ = 0.10 mm^−^^1^
α = 83.92 (3)º	*T* = 113 (2) K
β = 80.21 (3)º	Block, yellow
γ = 64.94 (3)º	0.20 × 0.18 × 0.12 mm
*V* = 773.0 (3) Å^3^	

### Data collection

**Table:**

Rigaku Saturn CCD area-detector diffractometer	3528 independent reflections
Radiation source: rotating anode	2679 reflections with *I* > 2σ(*I*)
Monochromator: confocal	*R*_int_ = 0.034
Detector resolution: 7.31 pixels mm^-1^	θ_max_ = 27.5º
*T* = 113(2) K	θ_min_ = 2.5º
ω and φ scans	*h* = −9→9
Absorption correction: multi-scan(SADABS; Sheldrick, 1996)	*k* = −9→9
*T*_min_ = 0.980, *T*_max_ = 0.988	*l* = −21→21
12548 measured reflections	

### Refinement

**Table:**

Refinement on *F*^2^	Secondary atom site location: difference Fourier map
Least-squares matrix: full	Hydrogen site location: inferred from neighbouring sites
*R*[*F*^2^ > 2σ(*F*^2^)] = 0.036	H atoms treated by a mixture of independent and constrained refinement
*wR*(*F*^2^) = 0.109	*w* = 1/[σ^2^(*F*_o_^2^) + (0.0647*P*)^2^ + 0.0542*P*] where *P* = (*F*_o_^2^ + 2*F*_c_^2^)/3
*S* = 1.06	(Δ/σ)_max_ = 0.002
3528 reflections	Δρ_max_ = 0.31 e Å^−^^3^
220 parameters	Δρ_min_ = −0.25 e Å^−^^3^
Primary atom site location: structure-invariant direct methods	Extinction correction: none

### Special details

**Table:**

Experimental. ^1^H NMR (CD_3_OD:CDCl_3_, 5:1, δ, p.p.m.): 8.07 (d, 1H), 7.68 (t, 1H), 7.59 (t, 1H), 7.36 (d, 1H), 6.88 (d, 1H), 6.18 (d, 1H), 6.13 (s, 1H), 3.04 (s, 6H).
Geometry. All e.s.d.'s (except the e.s.d. in the dihedral angle between two l.s. planes) are estimated using the full covariance matrix. The cell e.s.d.'s are taken into account individually in the estimation of e.s.d.'s in distances, angles and torsion angles; correlations between e.s.d.'s in cell parameters are only used when they are defined by crystal symmetry. An approximate (isotropic) treatment of cell e.s.d.'s is used for estimating e.s.d.'s involving l.s. planes.
Refinement. Refinement of *F*^2^ against ALL reflections. The weighted *R*-factor *wR* and goodness of fit *S* are based on *F*^2^, conventional *R*-factors *R* are based on *F*, with *F* set to zero for negative *F*^2^. The threshold expression of *F*^2^ > σ(*F*^2^) is used only for calculating *R*-factors(gt) *etc*. and is not relevant to the choice of reflections for refinement. *R*-factors based on *F*^2^ are statistically about twice as large as those based on *F*, and *R*- factors based on ALL data will be even larger.

### Fractional atomic coordinates and isotropic or equivalent isotropic displacement parameters (Å^2^)

**Table:**

	*x*	*y*	*z*	*U*_iso_*/*U*_eq_	
O1	0.70462 (14)	−0.28138 (12)	0.19780 (5)	0.0243 (2)	
H1	0.651 (2)	−0.228 (2)	0.2486 (10)	0.036*	
O2	0.54875 (13)	−0.02370 (12)	0.31294 (5)	0.0228 (2)	
O3	0.88960 (13)	0.13445 (13)	0.30456 (5)	0.0267 (2)	
O4	0.83287 (14)	0.32743 (14)	0.40966 (6)	0.0345 (2)	
H4	0.973 (3)	0.242 (3)	0.4146 (10)	0.052*	
O5	0.21609 (13)	0.11128 (13)	0.44162 (5)	0.0245 (2)	
H5	0.322 (3)	0.069 (2)	0.4029 (10)	0.037*	
N1	0.81331 (15)	−0.03715 (16)	−0.07467 (6)	0.0245 (2)	
C1	0.61529 (16)	0.07774 (16)	0.17518 (7)	0.0172 (2)	
C2	0.60924 (17)	0.23363 (17)	0.11636 (7)	0.0192 (2)	
H2	0.5611	0.3674	0.1342	0.023*	
C3	0.66995 (18)	0.19999 (18)	0.03453 (7)	0.0211 (2)	
H3	0.6602	0.3099	−0.0032	0.025*	
C4	0.74787 (17)	−0.00015 (18)	0.00592 (7)	0.0203 (2)	
C5	0.75624 (17)	−0.15813 (17)	0.06392 (7)	0.0206 (2)	
H5A	0.8070	−0.2923	0.0461	0.025*	
C6	0.69205 (17)	−0.12142 (16)	0.14605 (7)	0.0184 (2)	
C7	0.7924 (2)	0.1273 (2)	−0.13552 (7)	0.0286 (3)	
H7A	0.6454	0.2228	−0.1325	0.043*	
H7B	0.8422	0.0720	−0.1902	0.043*	
H7C	0.8754	0.1975	−0.1247	0.043*	
C8	0.9050 (2)	−0.2431 (2)	−0.10245 (8)	0.0283 (3)	
H8A	1.0167	−0.3284	−0.0704	0.042*	
H8B	0.9627	−0.2441	−0.1604	0.042*	
H8C	0.7975	−0.2956	−0.0953	0.042*	
C9	0.54677 (17)	0.11448 (17)	0.26072 (7)	0.0177 (2)	
C10	0.45044 (17)	0.32664 (16)	0.29110 (6)	0.0165 (2)	
C11	0.24430 (18)	0.44749 (17)	0.28008 (7)	0.0216 (2)	
H11	0.1761	0.4021	0.2476	0.026*	
C12	0.13725 (18)	0.63378 (17)	0.31616 (7)	0.0228 (3)	
H12	−0.0030	0.7156	0.3079	0.027*	
C13	0.23498 (18)	0.70024 (17)	0.36419 (7)	0.0226 (3)	
H13	0.1615	0.8270	0.3893	0.027*	
C14	0.44038 (18)	0.58132 (17)	0.37548 (7)	0.0204 (2)	
H14	0.5068	0.6272	0.4086	0.024*	
C15	0.55075 (17)	0.39511 (16)	0.33878 (6)	0.0168 (2)	
C16	0.77429 (17)	0.27111 (16)	0.34857 (7)	0.0185 (2)	
C17	0.2575 (2)	0.2154 (2)	0.49935 (7)	0.0287 (3)	
H17A	0.1589	0.3581	0.4986	0.043*	
H17B	0.4004	0.2052	0.4851	0.043*	
H17C	0.2418	0.1545	0.5541	0.043*	

### Atomic displacement parameters (Å^2^)

**Table:**

	*U*^11^	*U*^22^	*U*^33^	*U*^12^	*U*^13^	*U*^23^
O1	0.0321 (5)	0.0178 (4)	0.0208 (4)	−0.0080 (4)	−0.0025 (4)	−0.0044 (3)
O2	0.0310 (5)	0.0189 (4)	0.0175 (4)	−0.0093 (4)	−0.0025 (3)	−0.0021 (3)
O3	0.0226 (4)	0.0258 (4)	0.0254 (4)	−0.0014 (4)	−0.0052 (3)	−0.0090 (4)
O4	0.0214 (5)	0.0383 (5)	0.0411 (6)	−0.0025 (4)	−0.0116 (4)	−0.0211 (4)
O5	0.0207 (4)	0.0280 (5)	0.0251 (4)	−0.0089 (4)	−0.0022 (3)	−0.0083 (4)
N1	0.0215 (5)	0.0316 (6)	0.0169 (5)	−0.0071 (4)	−0.0011 (4)	−0.0064 (4)
C1	0.0155 (5)	0.0189 (6)	0.0173 (5)	−0.0062 (4)	−0.0030 (4)	−0.0042 (4)
C2	0.0170 (5)	0.0191 (5)	0.0203 (5)	−0.0059 (4)	−0.0017 (4)	−0.0045 (4)
C3	0.0186 (6)	0.0234 (6)	0.0191 (5)	−0.0068 (5)	−0.0020 (4)	−0.0008 (4)
C4	0.0127 (5)	0.0288 (6)	0.0175 (5)	−0.0054 (5)	−0.0023 (4)	−0.0063 (5)
C5	0.0179 (5)	0.0211 (6)	0.0220 (6)	−0.0054 (5)	−0.0028 (4)	−0.0083 (4)
C6	0.0161 (5)	0.0191 (6)	0.0205 (5)	−0.0063 (4)	−0.0047 (4)	−0.0032 (4)
C7	0.0255 (6)	0.0406 (8)	0.0170 (5)	−0.0111 (6)	−0.0017 (5)	−0.0027 (5)
C8	0.0265 (6)	0.0397 (7)	0.0223 (6)	−0.0161 (6)	0.0031 (5)	−0.0158 (5)
C9	0.0157 (5)	0.0188 (5)	0.0190 (5)	−0.0061 (4)	−0.0045 (4)	−0.0032 (4)
C10	0.0193 (5)	0.0161 (5)	0.0132 (5)	−0.0064 (4)	−0.0011 (4)	−0.0021 (4)
C11	0.0219 (6)	0.0232 (6)	0.0192 (5)	−0.0071 (5)	−0.0057 (4)	−0.0044 (4)
C12	0.0190 (6)	0.0215 (6)	0.0228 (6)	−0.0026 (5)	−0.0045 (4)	−0.0023 (5)
C13	0.0238 (6)	0.0170 (5)	0.0234 (6)	−0.0052 (5)	−0.0007 (5)	−0.0041 (4)
C14	0.0224 (6)	0.0184 (6)	0.0220 (5)	−0.0095 (5)	−0.0029 (4)	−0.0038 (4)
C15	0.0182 (5)	0.0169 (5)	0.0154 (5)	−0.0077 (4)	−0.0012 (4)	−0.0005 (4)
C16	0.0201 (5)	0.0181 (5)	0.0186 (5)	−0.0088 (5)	−0.0025 (4)	−0.0022 (4)
C17	0.0283 (6)	0.0366 (7)	0.0220 (6)	−0.0129 (6)	−0.0028 (5)	−0.0079 (5)

### Geometric parameters (Å, °)

**Table:**

O1—C6	1.3550 (14)	C7—H7A	0.9800
O1—H1	0.92 (2)	C7—H7B	0.9800
O2—C9	1.2561 (14)	C7—H7C	0.9800
O3—C16	1.2092 (14)	C8—H8A	0.9800
O4—C16	1.3240 (14)	C8—H8B	0.9800
O4—H4	0.94 (2)	C8—H8C	0.9800
O5—C17	1.4237 (15)	C9—C10	1.5075 (16)
O5—H5	0.87 (2)	C10—C11	1.3919 (16)
N1—C4	1.3569 (15)	C10—C15	1.4041 (15)
N1—C8	1.4537 (17)	C11—C12	1.3892 (17)
N1—C7	1.4587 (17)	C11—H11	0.9500
C1—C2	1.4105 (16)	C12—C13	1.3850 (17)
C1—C6	1.4258 (16)	C12—H12	0.9500
C1—C9	1.4367 (15)	C13—C14	1.3862 (17)
C2—C3	1.3699 (16)	C13—H13	0.9500
C2—H2	0.9500	C14—C15	1.3950 (16)
C3—C4	1.4281 (17)	C14—H14	0.9500
C3—H3	0.9500	C15—C16	1.4928 (16)
C4—C5	1.4101 (17)	C17—H17A	0.9800
C5—C6	1.3800 (16)	C17—H17B	0.9800
C5—H5A	0.9500	C17—H17C	0.9800
			
C6—O1—H1	105.2 (9)	N1—C8—H8C	109.5
C16—O4—H4	109.9 (10)	H8A—C8—H8C	109.5
C17—O5—H5	109.8 (10)	H8B—C8—H8C	109.5
C4—N1—C8	120.41 (11)	O2—C9—C1	122.52 (10)
C4—N1—C7	121.24 (10)	O2—C9—C10	116.45 (9)
C8—N1—C7	118.35 (10)	C1—C9—C10	120.82 (10)
C2—C1—C6	116.93 (10)	C11—C10—C15	119.40 (10)
C2—C1—C9	122.49 (10)	C11—C10—C9	117.74 (10)
C6—C1—C9	120.58 (10)	C15—C10—C9	122.31 (10)
C3—C2—C1	122.67 (10)	C12—C11—C10	120.65 (11)
C3—C2—H2	118.7	C12—C11—H11	119.7
C1—C2—H2	118.7	C10—C11—H11	119.7
C2—C3—C4	120.04 (11)	C13—C12—C11	120.03 (11)
C2—C3—H3	120.0	C13—C12—H12	120.0
C4—C3—H3	120.0	C11—C12—H12	120.0
N1—C4—C5	121.05 (11)	C12—C13—C14	119.83 (11)
N1—C4—C3	120.88 (11)	C12—C13—H13	120.1
C5—C4—C3	118.07 (10)	C14—C13—H13	120.1
C6—C5—C4	121.21 (11)	C13—C14—C15	120.80 (11)
C6—C5—H5A	119.4	C13—C14—H14	119.6
C4—C5—H5A	119.4	C15—C14—H14	119.6
O1—C6—C5	117.56 (10)	C14—C15—C10	119.28 (10)
O1—C6—C1	121.36 (10)	C14—C15—C16	120.61 (10)
C5—C6—C1	121.08 (11)	C10—C15—C16	120.10 (10)
N1—C7—H7A	109.5	O3—C16—O4	123.78 (11)
N1—C7—H7B	109.5	O3—C16—C15	123.12 (10)
H7A—C7—H7B	109.5	O4—C16—C15	113.10 (10)
N1—C7—H7C	109.5	O5—C17—H17A	109.5
H7A—C7—H7C	109.5	O5—C17—H17B	109.5
H7B—C7—H7C	109.5	H17A—C17—H17B	109.5
N1—C8—H8A	109.5	O5—C17—H17C	109.5
N1—C8—H8B	109.5	H17A—C17—H17C	109.5
H8A—C8—H8B	109.5	H17B—C17—H17C	109.5
			
C6—C1—C2—C3	1.11 (16)	C6—C1—C9—C10	−175.35 (9)
C9—C1—C2—C3	−178.47 (10)	O2—C9—C10—C11	−97.87 (12)
C1—C2—C3—C4	−1.53 (17)	C1—C9—C10—C11	76.97 (14)
C8—N1—C4—C5	−3.56 (16)	O2—C9—C10—C15	73.53 (14)
C7—N1—C4—C5	175.95 (10)	C1—C9—C10—C15	−111.62 (12)
C8—N1—C4—C3	175.95 (10)	C15—C10—C11—C12	−0.42 (16)
C7—N1—C4—C3	−4.54 (16)	C9—C10—C11—C12	171.25 (10)
C2—C3—C4—N1	−178.45 (10)	C10—C11—C12—C13	−0.52 (17)
C2—C3—C4—C5	1.08 (16)	C11—C12—C13—C14	0.63 (18)
N1—C4—C5—C6	179.25 (10)	C12—C13—C14—C15	0.20 (17)
C3—C4—C5—C6	−0.27 (16)	C13—C14—C15—C10	−1.13 (16)
C4—C5—C6—O1	179.85 (10)	C13—C14—C15—C16	177.71 (10)
C4—C5—C6—C1	−0.12 (17)	C11—C10—C15—C14	1.23 (16)
C2—C1—C6—O1	179.77 (9)	C9—C10—C15—C14	−170.04 (10)
C9—C1—C6—O1	−0.65 (16)	C11—C10—C15—C16	−177.62 (10)
C2—C1—C6—C5	−0.27 (16)	C9—C10—C15—C16	11.12 (15)
C9—C1—C6—C5	179.32 (10)	C14—C15—C16—O3	−162.76 (11)
C2—C1—C9—O2	178.74 (10)	C10—C15—C16—O3	16.06 (16)
C6—C1—C9—O2	−0.82 (17)	C14—C15—C16—O4	16.69 (15)
C2—C1—C9—C10	4.21 (16)	C10—C15—C16—O4	−164.48 (10)

### Hydrogen-bond geometry (Å, °)

**Table:**

*D*—H···*A*	*D*—H	H···*A*	*D*···*A*	*D*—H···*A*
O1—H1···O2	0.92 (2)	1.75 (2)	2.589 (2)	151 (1)
O4—H4···O5^i^	0.94 (2)	1.70 (2)	2.631 (2)	168 (2)
O5—H5···O2	0.87 (2)	1.95 (2)	2.815 (2)	178 (2)
C7—H7B···O3^ii^	0.98	2.54	3.463 (2)	156
C13—H13···O5^iii^	0.95	2.53	3.331 (2)	142

Symmetry codes: (i) *x*+1, *y*, *z*; (ii) −*x*+2, −*y*, −*z*; (iii) *x*, *y*+1, *z*.
